# Medial Migration of a Broken Trochanteric Cable

**DOI:** 10.1155/2018/4590105

**Published:** 2018-08-05

**Authors:** Bedri Karaismailoglu, Turgut Nedim Karaismailoglu

**Affiliations:** ^1^Orthopedics and Traumatology Department, Istanbul University Cerrahpasa Medical Faculty, Kocamustafapasa Cad. No. 53 Fatih, Istanbul, Turkey; ^2^Orthopedics and Traumatology Department, Ondokuzmayıs University, Atakum, Samsun, Turkey

## Abstract

**Background:**

Cerclage wires or cables are commonly being used in hip reconstruction procedures like primary (especially in patients with developmental hip dysplasia) or revision arthroplasty. Local or distant migration of a broken cable or wire is a complication that might lead to devastating situations depending on the route of migration.

**Case Presentation:**

We report a case of a 40-year-old female who underwent bilateral total hip arthroplasty surgery due to bilateral developmental hip dysplasia. Trochanteric osteotomy was needed in her right hip to advance trochanter major distally. Trochanteric fixation was achieved by a cerclage cable system. Four years after the surgery, the patient referred to the hospital with a prominence and pain in her right inguinal area. Radiographies revealed medial migration of a broken trochanteric cable part. The possible route of the cable was through medial adductor muscles, posterior to the femoral neurovascular bundle and anterior to the sciatic nerve. Both migrated and remaining parts of the cable were extracted under general anesthesia.

**Conclusion:**

Broken cables should be followed carefully due to their sharpness and possible serious complications secondary to distant migration. Extraction might be inevitable in case of a close relation with neurovascular structures.

## 1. Introduction

Cerclage cables have been used widely in fixation of fractures and osteotomies during reconstruction procedures. Even if it is rare, local or distant migration of broken cables can be experienced. While broken cables might cause localized symptoms like soft tissue irritation and pain, other serious complications might occur due to distant migration. In this case report, we report a broken trochanteric cable part which migrated medially to a subcutaneous place in the inguinal area and caused a prominence with pain. To our knowledge, no similar route for a broken cable was reported in the literature.

## 2. Case Presentation

**Figure 1:**
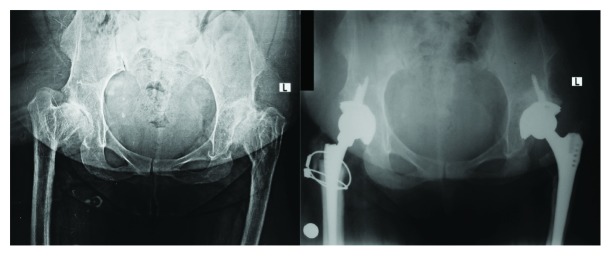
Preoperative and postoperative 2-year pelvis anteroposterior radiographs of the patient.

**Figure 2:**
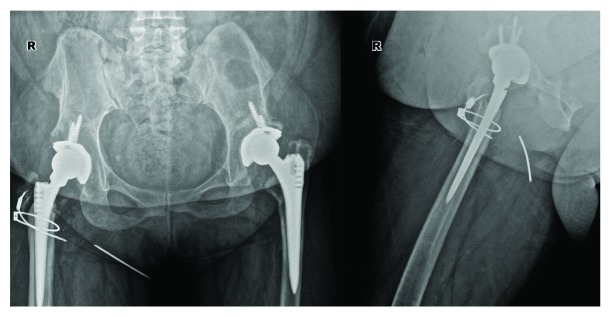
Postoperative 4-year pelvis anteroposterior and hip lateral radiographs of the patient showing medial migration of the broken cable part.

**Figure 3:**
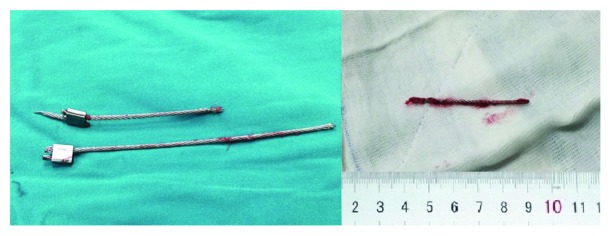
Broken and remaining parts of the cable.

A 40-year-old female patient presented with pain and decreased range of motion in her both hips. She was unable to maintain her daily activities. Radiographies revealed bilateral osteoarthritis of the hip, secondary to developmental hip dysplasia, and the patient underwent bilateral total hip arthroplasty (Figure 1). In her right hip, trochanteric osteotomy was needed to restore hip biomechanics and the fixation was made by a stainless steel cable system. After a 4-year pain-free period of time, she presented with pain and tenderness in her right inguinal area since last month. There was an immobile and painful prominence by palpation. The pain was not related to weight bearing, and hip examination did not reveal any pathology. In routine radiographies, it was discovered that the trochanteric cable was broken and approximately 5 cm part of the cable migrated medially (Figure 2). The patient's medical records were investigated and it was found out that her last radiography was taken 2 years before, in which the cable was firm and one part. The patient had not returned for follow-up since that date. C-reactive protein, blood counts, and sedimentation rate were between reference values, and there was no clinical sign of infection. The broken part of the cable was extracted with a small incision from the medial, while the remaining part was extracted with a larger lateral incision under general anesthesia (Figure 3). The tip of the broken cable part was buried in medial adductor muscles which lies posterior to the femoral neurovascular bundle and anterior to the sciatic nerve. The patient was comfortable and pain-free at her follow-ups after extraction.

## 3. Discussion

Trochanteric osteotomy is one of the important parts of a lateral approach in high hip arthroplasty or revision [1]. Besides removing the old component, it is a method providing easier canal preparation and making it possible to move trochanter distally in high hips. Fixation of the trochanter is still an important challenge for the surgeons. Monofilament wires, multifilament cables, or grip systems can be used in trochanteric fixation.

All trochanteric fixation methods have their own advantages and disadvantages. Monofilament wires produce less debris compared to the multifilament cable constructs, but kinking of the wires decreases their strength and makes them vulnerable to wire breakage, trochanteric nonunion, or migration [2]. These disadvantages and inadequate mechanical properties of wires led to the development of new constructs [3].

Multifilament cables are another option for trochanteric fixation, and their mechanical properties are found to be superior compared to the wires in some biomechanical and clinical studies [4, 5]. Their multifilament structure prevents kinking, and their application is easier compared to the wires. Cable tensioning provides better compression at the osteotomy site [2]. However, the multifilament structure of the cables leads to fragmentation and debris generation significantly more than wires, which can result in third-body polyethylene wear and revision surgery [6].

Cable grip systems are preferred mostly due to their superior mechanical properties like better resistance to fatigue and higher yield and breaking strength [6, 7]. But their bulky structure causes symptoms like pain and bursitis more often than other constructs [8]. Wire or cable breakage is also an important complication which might lead to trochanteric nonunion and revision surgery. Although there are some conflicting reports, the breakage rates of a cable grip construct were generally reported lower compared to the wire constructs [2].

Another important but rare complication of these constructs is hardware migration. Some cases have been reported in the literature related to cable or wire failure and their local and distant migration [9–11], but actual prevalence is not well-known. In a study, Ritter et al. found no difference between wires and cables in the possibility of breakage [12], but migrations were mostly reported with wires rather than cables, probably due to multifilament structures of cables limiting their advance in a soft tissue. Broken wire or cable parts can be followed up with direct radiographs, and mostly no intervention is needed when there is no symptom.

After failure, broken parts of the implants can migrate locally or distantly. Local migration to subcutaneous place might cause local irritation around the area of surgery. Broken implant or debris might migrate to intra-articular place and lead to loosening or implant wear. In some cases, distant migration can occur and serious complications like vessel, ureter, rectum, vagina, and uterus injuries might take place. Migrations happen usually through soft tissues, but some cases through the circulation system also have been reported, causing cardiopulmonary problems and even death [10, 13, 14].

In our case, medial migration of the cable part and its possible route between sciatic nerve and femoral neurovascular bundle were the distinctive features. To our knowledge, no similar route for a broken cable is reported. Fortunately, it reached subcutaneous tissue and caused only mild clinic symptoms.

When cables are used as a fixation device, a closer follow-up of the patients might be needed if the cable is broken and prone to migration. To predict or prevent possible distant migration in asymptomatic cases, a couple of factors should be considered. Age and activity of the patient; size, shape, sharpness, and bluntness of the broken cable; and possible relation to important anatomic structures are important to decide for prophylactic surgical extraction. When possible arterial, venous, or nervous system and organ injuries due to this immigration are considered, true early diagnosis and early treatment of these migrations are essential.

## Additional Points

*Authorship Declaration.* All authors listed meet the authorship criteria according to the latest guidelines of the International Committee of Medical Journal Editors, and all authors are in agreement with the manuscript.

## References

[1] Browne A. O., Sheehan J. M. (1986). Trochanteric osteotomy in Charnley low-friction arthroplasty of the hip. *Clinical Orthopaedics and Related Research*.

[2] Jarit G. J., Sathappan S. S., Panchal A., Strauss E., di Cesare P. E. (2007). Fixation systems of greater trochanteric osteotomies: biomechanical and clinical outcomes. *The Journal of the American Academy of Orthopaedic Surgeons*.

[3] Dall D., Miles A. (1983). Re-attachment of the greater trochanter. The use of the trochanter cable-grip system. *The Journal of Bone and Joint Surgery, British Volume*.

[4] MacDonald S. J., Cole C., Guerin J., Rorabeck C. H., Bourne R. B., McCalden R. W. (2003). Extended trochanteric osteotomy via the direct lateral approach in revision hip arthroplasty. *Clinical Orthopaedics and Related Research*.

[5] Shaw J. A., Daubert H. B. (1988). Compression capability of cerclage fixation systems. A biomechanical study. *Orthopedics*.

[6] Silverton C. D., Jacobs J. J., Rosenberg A. G., Kull L., Conley A., Galante J. O. (1996). Complications of a cable grip system. *The Journal of Arthroplasty*.

[7] Hersh C. K., Williams R. P., Trick L. W., Lanctot D., Athanasiou K. (1996). Comparison of the mechanical performance of trochanteric fixation devices. *Clinical Orthopaedics and Related Research*.

[8] Baril Y., Bourgeois Y., Brailovski V., Duke K., Laflamme G. Y., Petit Y. (2013). Improving greater trochanteric reattachment with a novel cable plate system. *Medical Engineering & Physics*.

[9] Makki D. Y., Goru P., Prakash V., Aldam C. H. (2011). Migration of a broken trochanteric wire to the popliteal fossa. *The Journal of Arthroplasty*.

[10] Marchi E., Reis M. P., Carvalho M. V. (2008). Transmediastinal migration of Kirschner wire. *Interactive Cardiovascular and Thoracic Surgery*.

[11] Fong Y.-C., Hsu H.-C., Lin W.-C. (2005). Intrapelvic migration of a Kirschner wire. *Journal of the Chinese Medical Association*.

[12] Ritter M. A., Lutgring J. D., Davis K. E., Berend M. E., Meding J. B. (2006). A clinical, radiographic, and cost comparison of cerclage techniques: wires vs cables. *The Journal of Arthroplasty*.

[13] Lyons F. A., Rockwood C. A. (1990). Migration of pins used in operations on the shoulder. *The Journal of Bone & Joint Surgery*.

[14] Fuster S., Palliso F., Combalia A., Sanjuan A., Garcia S. (1990). Intrathoracic migration of a Kirschner wire. *Injury*.

